# Roles and practices of general practitioners and psychiatrists in management of depression in the community

**DOI:** 10.1186/1471-2296-7-5

**Published:** 2006-01-30

**Authors:** Sophie Tardieu, Alain Bottero, Patrick Blin, Michael Bohbot, Sylvia Goni, Alain Gerard, Isabelle Gasquet

**Affiliations:** 1 Medical Evaluation Department, Assistance Publique-Hôpitaux de Marseille, Marseille, France; 2 Public Health Department, EA 3279, Faculté de Médecine, Marseille, France; 3 Paris; 4 Pharmacology Department, Université Victor Segalen Bordeaux 2, Bordeaux, France; 5 Lundbeck-France, Paris; 6 National Institute of Health and Medical Research, INSERM-U 669 Paris, France; 7 Direction de la Politique médicale, Assistance Publique – Hôpitaux de Paris, Paris, France

## Abstract

**Background:**

Little is known about depressed patients' profiles and how they are managed. The aim of the study is to compare GPs and psychiatrists for 1°) sociodemographic and clinical profile of their patients considered as depressed 2°) patterns of care provision.

**Methods:**

The study design is an observational cross-sectional study on a random sample of GPs and psychiatrists working in France. Consecutive inclusion of patients seen in consultation considered as depressed by the physician. GPs enrolled 6,104 and psychiatrists 1,433 patients. Data collected: sociodemographics, psychiatric profile, environmental risk factors of depression and treatment. All clinical data were collected by participating physicians; there was no direct independent clinical assessment of patients to check the diagnosis of depressive disorder.

**Results:**

Compared to patients identified as depressed by GPs, those identified by psychiatrists were younger, more often urban (10.5% v 5.4% – OR = 2.4), educated (42.4% v 25.4% – OR = 3.9), met DSM-IV criteria for depression (94.6% v 85.6% – OR = 2.9), had been hospitalized for depression (26.1% v 15.6% – OR = 2.0) and were younger at onset of depressive problems (all adjusted p < .001). No difference was found for psychiatric and somatic comorbidity, suicide attempt and severity of current depression.

Compared to GPs, psychiatrists more often prescribed tricyclics and very novel antidepressants (7.8% v 2.3% OR = 5.0 and 6.8% v 3.0% OR = 3.8) with longer duration of antidepressant treatment. GPs' patients received more "non-conventional" treatment (8.8% v 2.4% OR = 0.3) and less psychotherapy (72.2% v 89.1% OR = 3.1) (all adjusted p < .001).

**Conclusion:**

Differences between patients mainly concerned educational level and area of residence with few differences regarding clinical profile. Differences between practices of GPs and psychiatrists appear to reflect more the organization of the French care system than the competence of providers.

## Background

Depression is a highly prevalent disorder associated with enormous personal and societal cost [1] The global burden of mental illness is expected to rise sharply over the coming decades. The WHO Global Burden of Disease Study estimates that by 2020, major depression will rank as the second cause of disability[2] There is general agreement in European community studies that the current (12-month) prevalence rate for major depressive disorder is approximately 6%[3] Excluding hypertension, depression is commoner in primary care settings than any other condition. An average prevalence rate of 10.4% for current depressive episode (ICD-10) was reported by the WHO study of consecutive presenters in primary care[4]

In the health care system overall, GPs play a key role in diagnosing and treating patients with depressive disorders [5-7].

From the 1980s, numerous well-designed studies have reported that major depressive disorder is under-diagnosed and under-treated by GPs. [8-10] In comparison to the extensive literature on detection and diagnosis of depression, little is known about depressed patients' profiles and how they are actually treated outside the USA and the UK. To design quality improvement programs, information is urgently needed about current practices and the reasons for variation in "real life" settings.

The present observational study aimed to compare GPs and private psychiatrists 1°) for sociodemographic and clinical profile of their patients seen in consultation and considered as depressed by GPs (PDGPs) and psychiatrists (PDPs) 2°) for patterns of care provision for these patients.

## Methods

### Study design and sample

1,815 psychiatrists and 9,593 GPS were personally approached in their practices. They were randomly selected from a national database of 4,330 private psychiatrists and 51,421 GPs. Among these, 361 private psychiatrists and 2,570 GPs agreed to participate (participation rate 26.8% and 19.9%, respectively).

Over a 3-month inclusion period, psychiatrists were asked to include prospectively the first five consecutive patients considered as depressed seen in consultation and GPs were asked to include prospectively the first three consecutive patients identified as depressed. Inclusion criteria were: patients considered as depressed by the practitioner (this means no direct independent clinical assessment of the patient (by the physician in charge) to check the diagnosis of depressive disorder), age 18 plus, ongoing depressive disorder, and patients with episodes of depression at any stage of treatment. Patients were included whether or not the physician prescribed treatment for depression. (84.5% of participating psychiatrists and 84.6% of participating GPs included at least one patient). Between September 2002 and February 2003, these practitioners enrolled 1,433 and 6104 patients respectively. The mean number of patients enrolled was 4.8 (sd = 0.69) for psychiatrists and 2.5 (sd = 0.41) for GPs.

Three groups of practitioners were considered: 1°) eligible practitioners that included patients, 2°) eligible practitioners that did not include patients and 3°) eligible practitioners that did not participate to the study. The comparison of these three groups showed over-representation of female GPs, under-representation of physicians in the Paris region and over-representation of practitioners in western regions of France (for both GPs and psychiatrists), and the Mediterranean region (psychiatrists only) in group 1. No other differences were detected (Table 1).

**Table 1:** Description of the eligible practitioners

	Eligible GPs				Eligible psychiatrists			
	participants		non-participant		participants		non-participant	

	at least one patient	no patient included			at least one patient	no patient included		

	n = 2175	n = 395	N = 7021	P*	n = 305	n = 56	N = 1454	P*

Distribution within the type of practitioner (%)	22.7	4.1	73.2		16.8	3.1	80.1	
Female gender (%)	20.7	14.1	na	0.003	38.3	26.3	na	0.09
Regional distribution (%)				<0.001				<0.001
Paris, surrounding departments	9.7	13.2	14.8		21.0	22.4	27.5	
Paris basin	13.7	16.7	18.3		10.2	19.0	14.4	
East	11.5	10.4	12.2		8.9	8.6	12.6	
North-east	10.9	6.1	8.5		5.6	5.2	7.7	
North	6.2	6.1	6.3		6.9	6.9	3.3	
West	17.6	8.9	13.6		13.1	6.9	11.2	
South-west	15.7	19.7	12.3		14.4	10.3	10.1	
Mediterranean region	14.6	19.0	14.0		20.0	20.7	13.2	
Age (%)			na	0.16			na	0.46
40 years and less	20.8	16.7			11.1	5.7		
41–50 years	48.3	49.0			48.3	49.1		
50 years and more	30.8	34.3			40.6	45.3		
Mean # of years of private practice (sd)	17.4 (8.3)	17.7 (7.8)	na	0.51	16.1 (7.2)	15.6 (6.8)	na	0.67
Mean # of patient consultations per month (sd)	468.6 (210.1)	526.7 (216.5)	na	0.08	302 (240.0)	na	na	-
Proportion of depressed patient/overall patient of the physician			na	0.19		na	na	-
≤ 5	15.5	12.0			0.0			
6–10	43.5	32.0			2.0			
11–20	33.3	36.0			5.6			
21–30	4.3	12.0			12.3			
31–40	2.5	8.0			23.3			
41–50	0.5	0.0			20.9			
>50	0.3	0.0			35.9			

* chi2 test, comparison of participants having included at least one patient v participants not having included any patient v. non participants (if data available),

na: data non available

### Data collected

All clinical data were collected by participating physicians; there was no direct independent clinical assessment by the physician in charge of the patient to check the diagnosis of depressive disorder. The physicians recorded selected cases on anonymous patient record forms. Information gathered was divided into thee domains:

1) Patient socio-demographic variables: gender, age, marital status, area of residence, living arrangements, professional status, educational status, and income level.

2) Present and past clinical profile, using DSM-IV criteria for major depressive disorders [11] interspersed with other symptoms, and also alcohol dependency, bipolar disorder, anxiety disorder, suicide attempts, hospitalization for depressive problems, past episode of depressive disorder and age at first depressive episode. The severity of the depressive disorders was evaluated with the CGI-severity scale(.)[12] A list of significant stressful events that might contribute to the depressive episode was also proposed.

3) Type of care provided for the current depressive episode (psychotropics, psychotherapy, non conventional treatment, concomitant care by another health professional) and how long the patient had been in care.

According to the French ethics committee procedures, the protocol was submitted to the national medical association committee (CNOM) and to the national committee for protection of individual privacy (CNIL). Before including a patient, the physician was required to provide an information letter to obtain informed consent.

### Statistical analysis

Psychiatrists were compared to GPs using logistic regression adjusted on patient gender and age. For variables related to care patterns, analyses were also adjusted on CGI score. As it was not possible to consider the "centre" (practitioner) effect in the logistic regressions in these models (the variable "centre" is nested in the dependent variable "psychiatrist v. GP"), other analyses were previously conducted in order to detect any « cluster effect » since each practitioner included several patients. Two additional logistic models were used with each of the criteria (sociodemographic profile, clinical status, life events, and medical care). The only variable not tested was "geographic area" because it was nested in the variable "centre".

In the two models the criterion was the dependent (binary) variable with the following effects tested: age, gender and CGI-score for medical care, type of respondent (psychiatrists versus GPs). In the first model the variable "centre" (as a random effect) was added but not in the second model. The P values obtained for the variable "psychiatrist v. GP" on each model were compared. They were all very similar.

Because of the large sample, a small difference in percentage was statistically significant. Therefore odd-ratios equal to or higher than 2 or equal to or lower than 0.5 were retained as being clinically relevant, these values being classically used in epidemiological studies.

Statistical analysis was performed using SAS^® ^software (version 8.2).

## Results

### Sociodemographic profile (Table 2)

**Table 2:** Comparison of sociodemographic profile of GPs' depressed patient and psychiatrists' depressed patient (n = 7537)

	GPs' patients n = 6104 n (%)	Psychiatrists' patients n = 1433 n (%)	P value *
Age			<0.001
< 30 years	681 (11.2)	206 (14.4)	
31–40 years	1390 (22.90)	346 (24.2)	
41–50 years	1607 (26.5)	443 (31.0)	
51–60 years	1210 (19.9)	314 (21.9)	
> 60 years	1186 (19.5)	122 (8.5)	
Female gender	4367 (71.7)	1004 (70.1)	0.22
			OR [IC95] **
Marital status			
Single	779 (12.8)	270 (18.9)	1.0
Married, living with partner	3695 (60.9)	841 (58.8)	0.80 [0,68 – 0.94]
Separated or divorced Widowed	1598 (26.3)	319 (22.3)	0.77 [0,63 – 0,95]
Living Arrangement			
Living alone	1368 (22.5)	337 (23.5)	1.0
Living with 1 person	1568 (25.8)	386 (27.0)	1.01 [0,86 – 1,19]
Living with ≥ 2 persons	3137 (51.7)	708 (49.5)	0.80 (0,69 – 0,93]
Geographic area			
Rural (< 50000)	4164 (69.0)	742 (52.5)	1,0
Urban (>50000)	1544 (25.6)	523 (37.0)	1,89 [1.67 –2.15]
Paris region	326 (5.4)	148 (10.5)	**2.44 **[1.98 – 3.01]
Profession			
Never worked	521 (8.8)	99 (7.0)	**1.0**
Farmer	**213 (3.6)**	28 (2.0)	**0.86 [0.54 – 1.35]**
Trade, commerce, artisan	428 (7.2)	59 (4.2)	**0.78 [0.55 – 1.11]**
Managerial, l professional	711 (12.0)	290 (20.5)	**2.11 **[1.63 – 2.74]
Intermediate Professions)	1243 (21.0)	416 (29.3)	1.73 [1.35 – 2.21]
Executives	2032 (34.3)	434 (30.6)	1.10 [0.87 – 1.40]
Worker	769 (13.0)	92 (6.5)	0.64 [0.47 – 0.87]
Occupation			
Employed	3536 (58.3)	943 (65.8)	1.0
Unemployed	533 (8.8)	122 (8.5)	0.85 [0.69 – 1.05]
Retired	**1194 (19.7)**	141 (9.8)	0.55 [0.43 – 0.70]
Homemaker	518 (8.5)	92 (6.4)	0.72 [0.56 – 0.91]
Student	111 (1.8)	53 (3.7)	1.52 [1.06 – 2.17]
Other	170 (2.8)	82 (5.7)	1.94 [1.47 – 2.57]
Educational level			
Primary	926 (15.9)	82 (5.8)	1,0
Lower secondary	1854 (31.9)	353 (25.1)	1.94 [1.50 – 2.51]
Upper secondary	1564 (26.9)	376 (26.7)	**2.39 **[1.85 – 3.10]
Higher education	1477 (25.4)	598 (42.4)	**3.95 **[3.06 – 5.09]
Income			
Destitute	83 (1.4)	19 (1.3)	1,0
Low	1119 (18.5)	228 (16.0)	0.98 [0.58 – 1.65]
Moderate	3569 (58.9)	830 (58.2)	1.14 [0.69 – 1.89]
High	1291 (21.3)	350 (24.5)	1.41 [0.84 – 2.36]

In bold: statistically significant odds ratio (p < 0.05) and less than 0.5 or higher than 2.0

* Chi2 test

** GPs' patients are the reference, odd ratio adjusted on gender and on age as a quantitative variable

The PDPs were younger than the PDGPs (44.3 years, sd = 12.3 v. 47.9 years sd = 14.9, p = <.001). The biggest difference appeared in the over 60s with twice the proportion of these patients in the PDGP compared to the PDP group.

PDPs more often lived in urban areas than PDGPs. For patients living in the Paris region, the likelihood of consulting a psychiatrist rather than a GP was 2.5 times greater. The distance covered to get to the consultation was much greater for PDP than for PDGP, with 62.7% of PDPs taking less than half hour to reach the consultation v. 90.7% of PDGPs (p adjusted on gender and age <0.001).

Educational level was the most marked socio-demographic factor influencing choice of a psychiatrist rather than a GP. The likelihood of being in the PDP group increased with the level of education.

There was no clear relationship between gender, occupation, matrimonial status, living arrangements or income and the choice to consult a psychiatrist or a GP.

### Clinical profile (Table 3)

**Table 3:** Comparison of clinical status and past history between GPs' patients and psychiatrists' patients (n = 7537)

	GPs' patients n = 6104 n (%)	Psychiatrists' patients n = 1433 n (%)	OR [IC95]*
**Status at the time of the clinical diagnosis of the current depressive episode**			

DSM-IV diagnosis of MDD			
Yes	5223 (85.6)	1355 (94.6)	**2.91 [2.28 – 3.70]**
No	880 (14.4)	77 (5.4)	1.0
Number of symptoms of MDD on criterion A**			
5–6	2397 (45.9)	416 (30.7)	1.0
7–9	2826 (54.1)	939 (69.3)	1.90 [1.67 – 2.16]
CGI-severity			
Normal to moderately ill	1350 (22.8)	215 (15.1)	1.0
Markedly to among the most ill	4562 (77.2)	1207 (84.9)	1.67 [1.43 – 1.97]
**Psychiatric antecedents**			
Past depressive episode			
Yes	3068 (51.8)	836 (58.8)	1.43 [1.27 – 1.61]
No	2853 (48.2)	585 (41.2)	1.0
Hospitalization for depressive problem			
Yes	940 (15.6)	371 (26.1)	**2.03 [1.77 – 2.34]**
No	5076 (84.4)	1050 (73.9)	1.0
Suicide attempt			
Yes	665 (11.1)	258 (18.2)	1.73 [1.48 – 2.03]
No	5312 (88.9)	1161 (81.8)	1.0
Bipolar disorder			
Yes	295 (4.9)	107 (7.5)	1.70 [1.35 – 2.15]
No	5703 (95.1)	1311 (92.5)	1.0
Alcohol dependence			
Yes	613 (10.2)	137 (9.6)	0.93 [0.76 – 1.14)
No	5412 (89.8)	1284 (90.4)	1.0
Anxiety disorder			
Yes	4798 (79.5)	1050 (74.3)	0.76 [0.67 – 0.87]
No	1239 (20.5)	363 (25.7)	1.0
Current chronic somatic disorder ***			
Yes	2830 (47.5)	500 (35.8)	0.72 [0.63 – 0.83]
No	3123 (52.5)	898 (64.2)	1.0

In bold: statistically significant odds ratio (p < 0.05) and less than 0.5 or higher than 2.0

* GPs' patient are the reference, odd ratio adjusted on gender and on age as a quantitative variable

** Excluding patient with no MDD diagnosis. Criteria A are depressed mood (A1), anhedonia (A2), weight loss when no dieting or weight gain (A3), insomnia or hypersomnia (A4), psychomotor agitation or retardation (A5), fatigue or loss of energy (A6), feeling of worthlessness or guilt (A7), diminished ability to think or concentrate (A8), suicidal behavior (A9)

*** Neurological, cardiovascular, pulmonary, hepato-gastro-enterological, endocrinological and others (including cancer)

The probability of meeting DSM-IV criteria for major depressive disorder for patients identified as depressed was higher for psychiatrist consultation than for GP consultation (94.6% v. 86%, adjusted OR = 2.9). At the same time, past hospitalization was twice as frequent among PDPs as among PDGPs.

For all other indicators of long-standing psychiatric problems (previous suicide attempts, bipolar disorder, past depressive episodes) and of severity of the episode (CGI score) groups did not differ clearly.

Mean age at the first depressive episode was slightly younger for PDPs than for PDGPs (33.0 years sd = 11.9 v. 36.8 years sd = 13.9, p adjusted on gender and age = 0.0003).

There were non-significant differences for co-morbidity with anxiety, alcohol dependency and somatic problems.

### Role of stressful life events (Table 4)

**Table 4:** Comparison of risk factors for depression between GPs' patients and psychiatrists' patients (n = 7537)

	GPs' patients n = 6104 n (%)	Psychiatrists' patients n = 1433 n (%)	OR [IC95]*
Serious health problem			
Yes	642 (10.5)	128 (8.9)	1.0
No	5462 (89.5)	1305 (91.1)	0.96 [0.78 – 1.18]
Death of a person close			
Yes	822 (14.4)	165 (11.5)	1.0
No	5222 (85.6)	1268 (88.5)	0.86 [0.72 – 1.03]
Physical or sexual abuse			
Yes	164 (2.7)	46 (3.2)	1.0
No	5940 (97.3)	1387 (96.8)	1.01 [0.79 – 1.54]
Pregnancy-childbirth			
Yes	151 (2.5)	38 (2.7)	1.0
No	5953 (97.5)	1395 (97.3)	0.88 [0.61 – 1.27]
Divorce, separation or conflict with a partner			
Yes	1759 (28.8)	399 (27.8)	1.0
No	4345 (71.2)	1034 (72.2)	0.88 [0.78 – 1.01]
Loss of job, unemployment			
Yes	373 (6.1)	81 (5.7)	1.0
No	5731 (93.9)	1352 (94.3)	0.85 [0.66 – 1.09]
Conflict or harassment at work			
Yes	760 (12.5)	228 (15.9)	1.0
No	5344 (87.5)	1205 (84.1)	1.23 [1.05 – 1.45]
Overwork			
Yes	807 (13.2)	178 (12.4)	1.0
No	5297 (86.8)	1255 (87.6)	0.86 [0.73 – 1.02]
Precarious social situation			
Yes	173 (2.8)	28 (2.0)	1.0
No	5931 (97.2)	1405 (98.0)	0.65 [0.43 – 0.97]

* GPs' patients are the reference, odd ratio adjusted on gender and on age as a quantitative variable

None of the stressful events explored (death, abuse, unemployment, problem at work, difficulties with a partner, health problem) were significantly associated with the choice to consult a psychiatrist or a GP nor were other stressful situations possibly linked with increased risk of depression (recent pregnancy, precarious social situation).

### Care patterns (Table 5)

**Table 5:** Comparison of medical care of GPs' patients and psychiatrists' patients

	GPs' patients n = 6104 n (%)	Psychiatrists' patients n = 1433 n (%)	OR (1) [IC95]*
Time in care			
Less than 1 year	1157 (19.0)	911 (63.8)	**14.96 [12.37 – 18.10]**
1 to 5 years	1989 (32.7)	366 (25.6)	**3.51 [2.87 – 4.29]**
more than 5 years	2928 (48.2)	151 (10.6)	1.0
Current prescription of... ...antidepressant			
None	1418 (23.2)	213 (14.9)	1.0
One SSRI	4110 (67.3)	943 (65.8)	1.51 [1.28 – 1.78]
tricyclic	142 (2.3)	112 (7.8)	**5.03 **[3.73 – 6.77]
Another class	185 (3.0)	98 (6.8)	**3.77 **[2.81 – 5.06]
2 or more antidepressants	249 (4.1)	67 (4.7)	1.73 [1.26 – 2.37]
...anxiolytic or hypnotic			
Yes	2706 (44.3)	749 (52.3)	1.34 [1.19 – 1.50]
No	3398 (55.7)	684 (47.7)	1.0
...antipsychotic			
Yes	331 (5.4)	136 (9.5)	1.63 [1.31 – 2.03]
No	5773 (94.6)	1297 (90.5)	1.0
Psychotherapy **			
Yes	4406 (72.2)	1277 (89.1)	**3.07 **[2.57 – 3.67]
No	1698 (27.8)	156 (10.9)	1.0
Care also provided by another health professional			
Yes	1245 (20.4)	221 (15.4)	0.62 [0.53 – 0.73]
No	4859 (79.6)	1212 (84.6)	1.0
Alternative or non-conventional treatment ***			
Yes	540 (8.8)	34 (2.4)	**0.26 **[0.19 – 0.38]
No	5564 (91.2)	1399 (97.6)	1.0

In bold: statistically significant odds ratio (p < 0.05) and less than 0.5 or higher than 2.0

* GPs' patients are the reference, odd ratio adjusted on gender, on age as a quantitative variable and on the CGI-severity score

** Supportive therapy, psychoanalytically oriented therapy or cognitive-behavioral therapy

*** Homeopathy, acupuncture, herbal medicine or mesotherapy

The mean time lapse between the beginning of the current depressive episode and the index consultation was longer for PDPs than for PDGPs (7.1 months SD = 15.5 versus 3.8 months sd = 11.0, p adjusted on gender, age and GCI score <0.001).

Conversely, duration of medical follow up by the practitioners was shorter for PDPs than for PDGPs.

The mean duration of the index consultation was significantly greater for PDPs (31.3 minutes sd = 8.8) than for PDGPs (23.4 minutes sd = 8.5, p adjusted on gender, age and CGI <0.001).

A minority of patients did not receive antidepressants. SSRI was the class most often prescribed. Prescriptions of tricyclic and non-SSRI non-tricyclic antidepressants were both more frequent among PDPs than among PDGPs. Among patients treated with antidepressants, treatment was more longstanding for PDPs than for PDGPs. Treatment had been instated for over 6 months for 41.2% of PDPs and 25.7% of PDGPs (p adjusted on gender, age and CGI <0.001).

Prescription patterns for anxiolytics and antipsychotics (alone or in coprescription) were not significantly different in the two groups.

A large majority of patients were receiving some sort of psychotherapy with higher probability of being in psychotherapy for PDPs than for PDGPs. Regarding the different types of psychotherapy, whereas no-marked difference was observed between the groups in the frequency of supportive therapy (65.9% for PDPs versus 68.9 for PDGPs) and cognitive-behavioural therapy (6.1% for PDP v. 4.7% for PDGP), psychoanalytically-oriented therapy was 4 times more frequent among PDPs than among PDGPs (respectively 23.1% v 4.5%).

The likelihood of receiving alternative therapy was lower for PDPs than for PDGPs. Collaboration between practitioner and other health professionals was slightly more frequent for PDGPs than for PDPs.

## Discussion

### Main findings

Differences between patients considered as depressed treated in general practice and those treated by community psychiatrists related mainly to certain patient socio-demographic characteristics (education, area of residence), with few or no differences regarding clinical profile. This pinpoints the difficulty for depressed patients from lower income categories in rural areas in accessing psychiatric care in France.

If no marked difference was found between the two types of physician for antidepressants, psychotherapy as such was clearly more frequently prescribed among psychiatrist attenders and conversely non conventional treatment (homeopathy, acupuncture, herbal medicine or mesotherapy) was more frequent among GP patients.

Differences between the two patient groups and treatment patterns appear to reflect more the organization of the French care system than the competence of providers.

### Limitations

Three main limitations should be noted.

First, the participation rates of GPs (26.8%) and psychiatrists (19.9%) were low, and the samples non-representative in terms of regional distribution for both GPs and psychiatrists, and in terms of gender among GPs. This may have induced a selection bias with over-representation of physicians (especially GPs) with a particular interest and training in the care of depressive patients. This could lead to underestimating differences between GPs and psychiatrists with respect to patient profile and care patterns. However this does not question the existence of such differences

Second, the study design excluded depressed patients not considered as depressed by the physicians. Several studies have found that GPs often under-diagnose depression. [8-10] Several studies have shown that less severely depressed patients are less likely to be identified as such by GPs[13,14] This could have induced a bias via underestimation of differences in clinical severity between patients seen by GPs and psychiatrists.

Third, the method relied on psychiatrists and GPs independently selecting up to five or three patients respectively, and there was no direct clinical assessment of patients, so that the results must be suspected of being subject to possible selection bias. Clinical differences between the samples could be due to differences between psychiatrists and GPs in assessment or recording, rather than to true differences between patients.

### Differences between depressed patients seen by GPs and those seen by psychiatrists

Results agree with previous studies showing that attitudes toward the use of mental health services are affected by age, gender and educational status [15-18]. They show that educational status was an important factor influencing the choice by depressed subjects of consulting either a GP or a psychiatrist, the less educated patients being less inclined to use mental health services. This is surprising because in France, patients are free to refer directly to a psychiatrist whatever their socio-demographic and clinical characteristics, with good reimbursement of specialist fees.

This difference could be explained by reluctance of subjects with minor (non-psychotic) psychiatric problems to consult a psychiatrist because of social barriers. It is however much more probably related to the existence of large unprovided rural zones, and a concentration of the psychiatric offer in the more attractive large cities. Physicians in France are free to choose their practice location. It also emphasizes the saturation of the care offer in France, especially marked for psychiatric care for lack of psychiatrists, following a decrease in the number of psychiatrists trained in the last decade[19] This generates an increase in consultation fees disadvantaging poorer clients.

Regarding the clinical profiles of patients considered as depressed by GPs and by psychiatrists, the study did not reveal very marked differences, perhaps contrary to expectations However, the practitioners are very likely to have pinpointed and included the most « characteristic » patients, which means that the results need to be received with caution.

Even if GPs' patients considered as depressed tended to have less severe depressive symptoms, no significant differences were found for other indicators of severity (comorbidity, suicidal behaviour, recurrent depression). The traditional notion that GPs mainly treat "social depressive reactions" that are not "real depression" is clearly out-dated. They also spent more time in consultation with them than with their other non-depressed patients: 23 minutes against only 14–19 minutes for GP patients overall according to the Société Française de Médecine Générale.

### Treatment of depression

If the well-tolerated once-a-day doses of SSRI constituted the first-line prescriptions established by both types of prescribers [20,21], psychiatrists more often prescribed tricyclic antidepressants and "new antidepressants" than GPs, perhaps because psychiatrists more often see patients after failure of the first-line of treatment (i.e. SSRI) and therefore modify therapeutic strategy, prescribing other antidepressants[22,23] The longer time lapse since the beginning of antidepressant treatment for psychiatrist patients than for GP patients reinforces the probability of this hypothesis.

Even if anxiolytics are not recommended in routine treatment for depression [24], in practice they are nevertheless often prescribed, with no differences between GPs and psychiatrists, confirming the results of a recent European study[25]

Psychotherapy (mainly supportive) was unexpectedly frequent. This result is not concordant with the fact that in France most of the GPs are not psychotherapists themselves, nor with the infrequency of double follow-up by GP and mental health professional observed in this study. It is however probably because the term "supportive psychotherapy" is understood by most GPs as a consultation that includes a mix of careful listening and personal counseling of the patient. This type of consultation takes time and may also explain the relatively long duration of GP consultations. GPs take time to listen and they do consider this sort of consultation as therapeutic in itself for depressed patients, in the same way as more structured psychotherapy. It can be added that psychotherapy has proven its efficacy in the treatment of depression, either as sole therapeutic intervention or as an adjunct to pharmacological treatment [26-29]

Non-conventional treatments are infrequent among treatments prescribed by both GPs and psychiatrists[30,31] However, these types of therapy occurred four times more frequently when a GP was consulted than when a psychiatrist was consulted. Since GPs are often the first health professional contacted for a depressive problem, they probably deal with greater patient expectations for "non aggressive" treatment than do psychiatrists. As the placebo effect is very important in mental health problems [32-34], positive response to demands for non conventional treatment can be expected, even if the advantage of these treatments over placebos remains to be proved.

## Conclusion

Differences between patients mainly concerned educational level and area of residence with few differences regarding clinical profile. Differences between practices of GPs and psychiatrists appear to reflect more the organization of the French care system than the competence of providers.

## Competing interests

The author(s) declare that they have no competing interests.

## Authors' contributions

All authors conceived of the study and participated in its design and coordination and helped to draft the manuscript. All authors read and approved the final manuscript.

## Pre-publication history

The pre-publication history for this paper can be accessed here:


